# Rapid onset renal deterioration in an adult with silent ureteropelvic junction obstruction

**DOI:** 10.4103/0970-1591.45553

**Published:** 2009

**Authors:** Nicholas J. Hellenthal, Sasha A. Thomas, Roger K. Low

**Affiliations:** Department of Urology, University of California, Davis Medical Center, Sacramento, California, USA

**Keywords:** Adult, renal function, ureteral obstruction

## Abstract

We report a case of a rapid renal deterioration due to ureteropelvic junction obstruction (UPJO) in an asymptomatic woman with prior normal diuretic renography. This case illustrates “silent” renal obstruction and the inability of diuretic renography in detecting significant renal obstruction. This case may favor close surveillance of any adult patient with potential UPJO, especially those with underlying renal disease or solitary kidney.

## INTRODUCTION

Ureteropelvic junction obstruction (UPJO) is an uncommon condition in adults. UPJO is most commonly diagnosed during childhood with treatment required for any patient demonstrating renal deterioration, stone formation, infection, or pain. Not all patients with hydronephrosis require intervention, although the natural history of untreated UPJO is better understood in children than adults. We describe a woman who rapidly developed renal deterioration due to UPJO despite lacking symptoms and previous normal evaluation by diuretic renography.

## CASE REPORT

**Figure 1:**
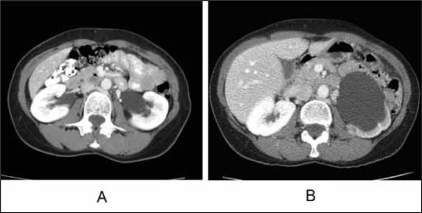
A. Initial CT scan. Note mild fullness of left renal pelvis. B. CT scan one year later. Note marked hydronephrosis of left kidney with thinning of renal parenchyma.

**Figure 2:**
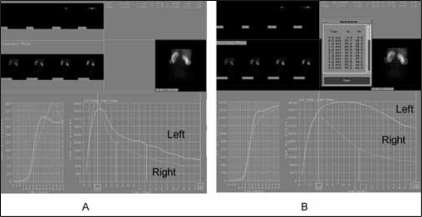
A. Initial MAG-3 renal scan. Note prompt bilateral washout after diuretic. B. MAG-3 renal scan one year later. Note delayed washout of tracer from the left kidney after furosemide.

A 62-year-old woman was referred for a brief episode of left flank pain and imaging demonstrating fullness of the left renal pelvis [Figure 1A]. She had no prior urologic history of colic, urolithiasis, or infection. Her serum creatinine was 1.0 mg/dl and urine culture was negative for bacteriuria. She was further evaluated with a MAG-3 diuretic renal scan demonstrating symmetric renal function and no evidence of left-sided obstruction (T ½ of 6 min) after administration of a diuretic [Figure 2A]. She was discharged from our clinic and instructed to return if she experienced further symptoms.

At the request of her primary care physician, the patient underwent repeat computed tomography (CT) imaging 12 months later despite being asymptomatic. Repeat imaging demonstrated severe left-sided hydronephrosis [Figure 1B]. Repeat nuclear renography at that time demonstrated loss of renal function from 50 to 10% [Figure 2B]. The patient then underwent placement of a left ureteral stent with no improvement in differential renal function on renography four weeks later. She subsequently elected for ureteral stent removal and has not had any repeated symptoms related to her UPJO at 6 months follow-up.

## DISCUSSION

The natural history of UPJO in adulthood is poorly understood but believed to be related to intrinsic narrowing of the upper ureter or extrinsic pressure on the ureter caused by aberrant vessels or fibrous bands.[1] As prenatal screening improves, adult patients account for a decreasing number of patients diagnosed with congenital UPJO.[2] Most adult patients with UPJO undergo treatment for symptoms related to pain, infection, or urolithiasis.

Although there is evidence that not all pediatric patients demonstrating hydronephrosis require intervention, the natural history of untreated UPJO in adults is less well documented. Most studies document preservation or improvement of renal function following treatment for adult UPJO.[3–5] A small study of 36 adult patients with unilateral hydronephrosis evaluated renal function following pyeloplasty versus strict observation.[6] Although a statistically significant increase in mean ipsilateral renal function on isotope renography after pyeloplasty was found, the study found no significant improvement in total glomerular filtration rate in the surgically treated group when compared to the untreated group at a mean of 35 months. They thus concluded that, in the absence of stone formation and infection, hydronephrosis in adults with two functioning kidneys is a benign condition and follow-up should be directed toward detecting these complications.

The case presented documents rapid renal deterioration from UPJO in an asymptomatic adult patient with prior normal evaluation for presence of obstruction. Although undoubtedly rare, this case illustrates that diuretic renography may not detect significant renal obstruction, and that renal deterioration may occur in an otherwise asymptomatic patient. The sensitivities of current standards for the diagnosis of UPJO, namely diuretic renography and CT scanning, are imperfect. There remains room for improvement in the diagnosis, perhaps at a functional imaging level or at a molecular level, of UPJO.

Nonetheless, serial imaging in all asymptomatic patients who have a normal evaluation for the presence of UPJO cannot be recommended, however repeat imaging (ultrasound or CT scanning) may be prudent in patients at risk for renal compromise (i.e., diabetes, uncontrolled hypertension, solitary kidney, etc.). Patients and physicians need be aware of silent renal obstruction and the potential for diuretic renography and CT scanning to, at least transiently, miss the diagnosis.
